# The Electro-Physiology of Man. With Practical Illustrations of New and Efficient Modes of Galvanic Treatment in a Variety of Cases

**Published:** 1844-01

**Authors:** 


ART. VII.—*The Electro-physiology of Man. With practical illustrations of new and efficient modes of Galvanic Treatment in a variety of cases.* By JOHN DODDRIDGE HUMPHREYS, Esq.—*London*, 1843. 12mo, pp. 228.

A MERE puff, designed to inform the public, that John Doddridge Humphreys, Esquire, practices medical galvanism in a new and superior style; curing patient after patient whose maladies have resisted all ordinary modes of treatment, and applying the most efficient remedies to almost all the diseases that flesh is heir to. As a scientific production it is totally valueless. The author's whole theory of the operation of electricity on the animal body is founded upon Dr. Wilson Philip's well-known experiments; the public faith in which, as identifying nervous agency with electricity, does not seem to be in the least shaken by the disproof with which this doctrine has been met by other experimentalists. We are far from discouraging the judicious application of electricity as a remedial means: since we regard it as a very important therapeutic agent, the true value of which has not yet been fairly tested. But assuredly our confidence in it will not be increased by any of the reports of John Doddridge Humphreys, Esquire, or of any other professed electrician; since we all know how much those who restrict themselves to any one particular plan of treatment, become prejudiced, even when their intentions are of the best possible character, in favour of its use. But we hope that our other great hospitals will follow the example of Guy's, and will add an electrical apparatus to their *armamentarium therapeuticum*, placing it under the most competent director who can be secured, and giving to the public an unbiassed report of the results of its application.

The true nature of Mr. Humphreys's book is shown by the fact, that he only indicates in the vaguest possible terms, in what his "new and efficient modes of galvanic treatment" consist; so that no one has the power of repeating his experiments, and of giving a more trustworthy account of their value.

